# 

**Published:** 1893-10-07

**Authors:** 


THE HOSPITAL.

PRICE TWOPENCE.

No. 367, Vol. XV.—Oct. 7th, 1893.

[Registered at the General Post Office as a Newspaper for transmission in the United Kingdom and Abroad.]

OCTOBER 7TH,

WITH EXTRA NURSING SUPPLEMENT

Per Annum, 8s. 6d., post free.

Monthly Parts, 6d.; post free, 8d.

Ditto per Annum, 8s., post free.

### THE HOSPITAL

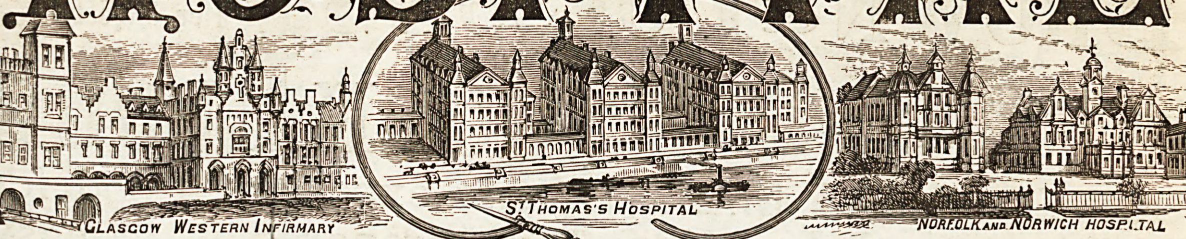

WITH EXTRA NURSING SUPPLEMENT.

No. 367. Vol. XV. SATURDAY, OCTOBER 7TH, 1893. [TWOPENCE.

